# The Role of Myeloid Cells in GBM Immunosuppression

**DOI:** 10.3389/fimmu.2022.887781

**Published:** 2022-05-31

**Authors:** Ya-Jui Lin, Caren Yu-Ju Wu, Janet Yuling Wu, Michael Lim

**Affiliations:** ^1^ Department of Neurosurgery, Chang Gung Medical Foundation, Linkou Medical Center, Taoyuan, Taiwan; ^2^ Department of Neurosurgery, Stanford University School of Medicine, Stanford, CA, United States; ^3^ Department of Neurosurgery, Chang Gung Medical Foundation, Keelung Chang Gung Memorial Hospital, Keelung, Taiwan

**Keywords:** glioma, myeloid cells, immunosuppression, immunotherapy, macrophage, neutrophil, dendritic cell

## Abstract

Gliomas are intrinsic brain tumors that originate from glial cells. Glioblastoma (GBM) is the most aggressive glioma type and resistant to immunotherapy, mainly due to its unique immune environment. Dimensional data analysis reveals that the intra-tumoral heterogeneity of immune cell populations in the glioma microenvironment is largely made up of cells of myeloid lineage. Conventional therapies of combined surgery, chemotherapy and radiotherapy have achieved limited improvements in the prognosis of glioma patients, as myeloid cells are prominent mediators of immune and therapeutic responses—like immunotherapy resistance—in glioma. Myeloid cells are frequently seen in the tumor microenvironment (TME), and they are polarized to promote tumorigenesis and immunosuppression. Reprogramming myeloid cells has emerged as revolutionary, new types of immunotherapies for glioma treatment. Here we detail the current advances in classifying epigenetic, metabolic, and phenotypic characteristics and functions of different populations of myeloid cells in glioma TME, including myeloid-derived suppressor cells (MDSCs), glioma-associated microglia/macrophages (GAMs), glioma-associated neutrophils (GANs), and glioma-associated dendritic cells (GADCs), as well as the mechanisms underlying promotion of tumorigenesis. The final goal of this review will be to provide new insights into novel therapeutic approaches for specific targeting of myeloid cells to improve the efficacy of current treatments in glioma patients.

## Introduction

Due to advanced diagnostic imaging tools including of computed tomography (CT) and magnetic resonance imaging (MRI), the incidence of brain tumors has increased recently (1). Brain tumors greatly affect the neurological function, psychological health, and quality of life of patients (1, 2). Gliomas are intrinsic tumors that originate from neuroglial progenitor cells. Glioblastoma (GBM), a grade IV glioma, is the most common primary malignant brain tumor (49.1%) with male predominance in United States (3). Based on the 2021 WHO classification, gliomas include adult-type diffuse gliomas, pediatric-type diffuse low-grade gliomas, pediatric-type diffuse high-grade gliomas, circumscribed astrocytic gliomas (4). Previously, glioblastomas were diagnosed based on the histologic findings of microvascular proliferation and/or necrosis and included both IDH-mutated (10%) and IDH wild-type (90%) tumors with very different prognoses. In WHO CNS5, GBMs will comprise only IDH wild-type tumors. Otherwise, IDH-mutant GBM is now referred to as IDH-mutant astrocytoma, WHO grade 4. In 2021, in response to modifications of diagnostic algorithms and mature results of many large clinical trials, the European Association of Neuro- Oncology (EANO) provide updated guidelines for the diagnosis and management of adult-type diffuse gliomas including GBMs (5). The standard of care for patients with GBM aged <70 years and with a KPS >70 is maximal resection with neurologic function preservation or biopsy followed by concurrent chemo-radiation and maintenance adjuvant chemotherapy (temozolomide, TMZ) (6). Elderly patients could be treated with low-dose radiotherapy or TMZ alone (7, 8). Once recurrence, no consensus for treatment is defined. Re-operation, radiotherapy (re-boost), nitrosourea regimens, TMZ re-challenges, and bevacizumab are all options, but benefit remained unclear on overall survival. On the other hand, recruitment into appropriate clinical trials should be considered when available. The new treatment modality, tumor-treating fields (TTF), demonstrated superior progression-free survival and overall survival outcomes in all patients and across all tumor subgroups when in addition to maintenance TMZ in patients with newly diagnosed GBM (9). However, the feasibility and cost-effectiveness of TTF are still concerned and remain controversial as a standard of care (10). In summary, the prognosis of GBM is still very poor, and effective therapies are urgently needed.

**Figure 1:**
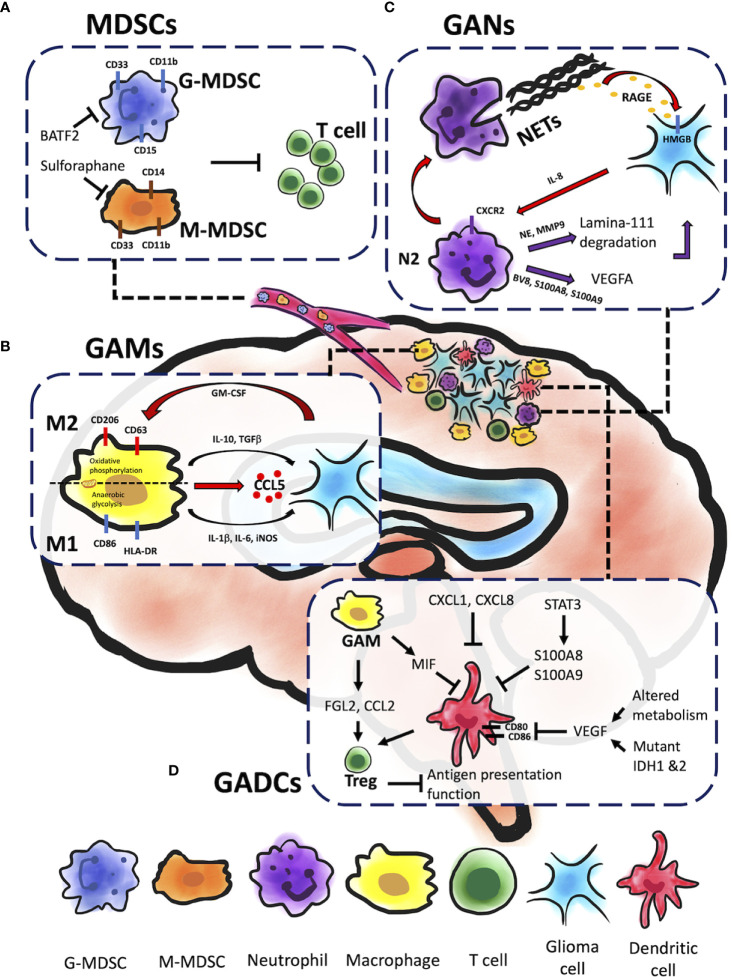
Myeloid cells within glioma microenvironment. Gliomas are composed of different types of myeloid immune cells which promote tumor progression, including MDSCs, GAMs, GANs, and GADCs. Each of these cell types contributes to glioma progression in unique ways. **(A)** Both G-MDSC and M-MDSC recruitments contribute to T cell inactivation and inhibition cytotoxicity of glioma cells. BATF-2 on G-MDSC and sulforaphane on M-MDSC could cause inhibitory effect and further prevent from T cell inactivation and glioma progression. **(B)** GAMs engage in significant bidirectional crosstalk with glioma cells. Glioma cells release cytokines and chemoattractants to recruit GAMs to the glioma microenvironment, and M2 GAMs in turn supply pro-tumorigenic and pro-survival factors. In addition, GM-CSF promote GAMs’ mitochondrial reprograming that sway between M1 and M2 inflammatory response leading to glioma resistance. **(C)** GANs can be reprogrammed to express pro-tumor phenotype (N2) with TGFβ signaling in the TME to facilitate tumor growth through NE and MMP9 secretion. The release of the pro-angiogenic factors BV8 and the S100 proteins (S100A8 and S100A9) by N2 GANs activate VEGFA to promote tumor growth. Glioma cells can induce NETs formation *via* IL-8 production. NETs are correlated with glioma progression and prognosis through a HMGB1/RAGE/IL-8 axis. **(D)** A variety of signaling molecules alter GADC migration, infiltration of the TME, maturation, and function. FGL2 and CCL2 secreted by GAMs and GADCs induce Treg activity, which suppresses antigen presentation function of GADCs. MIF, also secreted by GAMs, inhibits GADC maturation as well as migration and infiltration to the TME. The STAT3 signaling pathway inhibits GADC maturation, as does VEGF through inhibition of costimulatory factors CD80 and CD86. VEGF is expressed by tumor cells and influenced by mutant IDH1 and IDH2, as well as altered metabolism in the TME.

The aim of cancer immunotherapy is to overcome tumor immune resistance to promote tumor eradication. This strategy has demonstrated great progress and excellent results in recent years, especially since immune checkpoint inhibitors (ICIs) in melanoma and lung cancer (11). However, recent clinical trials of ICIs and vaccine therapies have shown negative results in GBM patients (12). Several obstacles, which include natures of heterogeneity and low mutation burden, and local/systemic immunosuppressive microenvironment, impede the success to GBM immunotherapies (13). Therefore, the tumor microenvironment (TME) is emerging as a critical regulator of cancer progression in glioma. Besides cancer cells, there are many different noncancerous cell types residing in TME, including endothelial cells, pericytes, fibroblasts, and immune cells (14). There is mounting evidence, however, that the TME alters myeloid cells—the most abundant nucleated hematopoietic cells in the human body—by converting them into potent immunosuppressive cells, including myeloid-derived suppressor cells (MDSCs), tumor-associated macrophages and microglia (TAMs), tumor-associated neutrophils (TANs), and tumor-associated dendritic cells (TADCs) (15). Here, we review the current understanding of the roles of myeloid-derived suppressor cells (MDSCs), glioma-associated macrophages (GAMs), glioma-associated neutrophils (GANs), and glioma-associated dendritic cells (GADCs) (**Table 1**). By developing a comprehensive understanding of the complex interactions of myeloid cells in glioma TME (**Figure 1**), we will greatly expand the range of therapeutic strategies available to target GBM, a devastating disease.

**Table 1:** Summary of tumor promotion function in myeloid cells.

Cell	Origin	Surface marker		Tumor promoting features
**MDSC**	Myeloblast (bone marrow)	G-MDSC	CD11b^+^CD14^-^CD33^+^HLA-DR^low/-^CD15^+^ (or CD66^+^)	• Loss of MHC class II from MDSCs interferes with T-cell mediated immune responses, leading to immunosuppressive TME.
	Monocyte/macrophage and dendritic cell precursor (bone marrow)	M-MDSC	CD11b^+^CD14^+^CD33^+^HLA-DR^low/-^CD15^-^	
**GAM (Glioma-associated macrophage)**	CNS resident microglia (York sac)	CD11b^+^CD45^low/int^ or CD11b^+^CD206^low/-^CD163^-^		• Mediate immunosuppression by upregulation of Arg-1, IL-10, TGF-β, CD206, CD163, CCL17, and CCL22, inhibitory immune checkpoints (PD-1, CTLA-4 and TIM-3).
	monocyte/macrophage and dendritic cell precursor (bone marrow)	CD11b^+^CD45^hi^ or CD11b^+^CD206^hi^CD163^+^		
**GAN (Glioma-associated neutrophil)**	Myeloblasts (bone marrow)	N1	CD66b^+^, CD11b^+^, CD101^+^, CD170^low^, CD54^+^, HLA-DR^+^, CD86^+^, CD15^high^	• N2 GANs suppress T cell immunity and induce genetic instability, tumor cell proliferation, angiogenesis, and metastasis • NETs induce the IL-8 expression, which is correlated with tumor burden and prognosis through a HMGB1- and RAGE/ERK/NF-kB axis-dependent manner.
		N2	CD66b^+^, CD11b^+^, CD170^high^, PDL1
**GADC (Glioma associated dendritic cell)**	monocyte/macrophage and dendritic cell precursor (bone marrow)	cDC	CD45^hi^, CD11b^+^, CD11c^+^, CD103^+^, CD205^+^, MHC II^+^	• FGL2 and CCL2 induce Treg to inhibit antigen presentation • MIF decreased GADC migration and maturation • Inhibit GADC maturation by STAT3 signaling pathway • Inhibit costimulatory factors CD80 and CD86 by VEGF, expressed VEGF is expressed by tumor cells and influenced by mutant IDH1 and IDH2

## Myeloid-Derived Suppressor Cells (MDSC)

Myeloid-derived suppressor cells (MDSCs) are a heterogeneous group of bone marrow-derived immature myeloid cells comprising of either monocytic or granulocytic at different stages of differentiation (16). There are three major types of MDSCs: granulocytic or polymorphic nuclear MDSCs (G/PMN-MDSCs), mononuclear MDSCs (M-MDSCs), and early-stage MDSCs (eMDSCs). Human G-MDSCs are characterized as CD11b^+^CD14^-^CD33^+^HLA-DR^low/-^CD15^+^ (or CD66^+^), M-MDSCs as CD11b^+^CD14^+^CD33^+^HLA-DR^low/-^CD15^-^, and eMDSCs as Lin^-^ (CD3^-^, CD14^-^, CD15^-^, CD19^-^, CD56^-^, HLA-DR^-^, and CD33^+^) (17). In healthy conditions, immature myeloid cells (IMCs) quickly differentiate into mature macrophages, granulocytes, or dendritic cells (DCs). Under pathological conditions such as glioma, inflammatory conditions prevent immature myeloid cells differentiation into mature myeloid cells resulting in MDSC accumulation (18). For example, IMCs from tumor-bearing mice had a significantly higher level of reactive oxygen species (ROS) than from tumor-free mice. Hydrogen peroxide (H_2_O_2_) but not superoxide radical anions were found to be the major component of ROS that prevents MDSCs differentiation of antigen-presenting cells (18). In human cancer patients, MDSCs are identified as HLA-DR^-^CD11b^+^CD14^-^CD33^+^ cells that co-express the myeloid differentiation markers, CD11b and CD33, while lacking mature lymphoid and myeloid cell markers, such as HLA-DR, an MHC class II molecule (19). This MHC class II molecule is normally found on antigen-presenting cells (APCs) and regulated by CIITA, a transactivator (20). Loss of MHC class II from MDSCs interferes with T-cell mediated immune responses, leading to immunosuppressive TME, and is correlated with poor clinical outcomes in glioma (20–22). Accumulating evidence has shown that glioma-released factors promote the recruitment of MDSCs, inhibiting T cell proliferation and leading to glioma growth (23). Sulforaphane treatment has been shown to prevent transformation of normal monocytes to M-MDSCs (23), and BATF2 inhibitor was shown to prevent glioma progression by inhibiting G-MDSCs recruitment (**Figure 1A**) (24). Immune checkpoints such as TIGIT, CTLA-4, PD-1 on T cells are thought to cause T cell exhaustion and associate with glioma recurrence (25, 26). Dual treatment of anti-PD-1 and anti-TIGIT was shown to increase effector T cell function and downregulate suppressive regulatory T cells (Tregs). However, a recent phase I clinical trial has revealed although neoadjuvant PD-1 blockade increases T cell function, these cells eventually transit into an exhausted stated and are inhibited by the myeloid suppressor population (25). A preclinical study also revealed sexual dimorphism: M-MDSCs were enriched in the male tumors whereas G-MDSCs were elevated in the females’ peripheral blood, both of which can be leveraged for therapeutic management (27).

## Glioma-Associated Microglia/Macrophages (GAMs)

The central nervous system (CNS) is considered to be immune-privileged environment. The blood-brain barrier (BBB) prevents activated T cells from entering CNS under steady-state and healthy conditions. Diseased states, such as glioma, cause BBB leaks, leading to immune cell infiltration from the periphery (28). However, such a belief has been amended as mouse and human studies revealed that tissue CD4^+^ and CD8^+^ cells patrol in the cerebrospinal fluid or brain parenchyma and can interact with ACPs (29, 30). Brain CD8^+^ T cells that were CD103^+^ associated with increased expression of tissue-homing chemokine receptors compared to those that were CD103^-^ (29), and CXCL12 was shown to promote T cell transmigration across BBB (31). Microglia, the major APC subset within the CNS, are functionally compromised in the glioma microenvironment, which decreases the effectiveness of tumor eradication at the initial stage, as well as later T-cell-dependent immune responses (20). Last but not least, microglia exhibit suppression of MHC class II (MHC-II) molecules, which limits T cell-dependent antitumor immunity (20, 22). The MHC-II molecules were thought to mediate antigen presentation whereas the mechanism of antigen presentation is complicated. Although MHC-II was muted in GAMs, this may be just a compiled factor contributing to GAM poorly activate T cells (32). In addition, Toll-like receptor 2 (TLR2) activation is prevailing found in GAMs that can downregulate MHC class II molecules in GAMs and prevents T cell proliferation and activation (20). The study has shown that glioma induces chronic inflammation in microglia and activates Toll-like receptor 2 (TLR2), triggering downstream MAPK/ERK signaling, and responses associated to loss of histone H3 acetylation at CIITA promoters (20). In the glioma microenvironment, various endogenous TLRs ligands, such as heat shock proteins, high-mobility-group box 1 (HMBG1), and damage-associated molecular patterns (DAMPs), are upregulated by necrotic cells. This upregulation is correlated with CIITA inhibition, contributing to glioma immune evasion (33, 34).

GAMs belong to myeloid lineages that are defined as CNS resident microglia and bone marrow-derived macrophages; they populate the TME and promote tumor progression (35). GAMs are the most prominent cell sub-type of the tumor mass (~30-50%). Tumor size is positively correlated to the number of GAMs shown to inversely correlate with overall survival in patients with recurrent GBM (35–37). Glioma-derived Granulocyte-macrophage colony-stimulating factor (GM-CSF) promotes activation of GAMs and production of CCL5 (**Figure 1B**), which further induce a series of calcium-dependent pathways such as p-PYK2 and p-CAMKII that lead to glioma progression (35). Generally, microglia are recognized as CD11b^+^CD45^low/int^ or CD11b^+^CD206^low/-^CD163^-^, whereas macrophages are recognized as CD11b^+^CD45^hi^ or CD11b^+^CD206^hi^CD163^+^ (38, 39). Common activation markers observed in microglia and macrophages include CD68, CD86, CD45, CX3CR1, and HLA-DR, though TMEM119, P2RY12 and CD49D (encoded by ITGA4) expression levels are higher in microglia and macrophages in the human brain (39–41). GAMs express molecules associated with M2 anti-inflammatory phenotype in mouse GBM models that include upregulation of Arginase-1 (Arg-1), IL-10, transforming growth factor-β (TGFβ), CD206, CD163, CCL17, and CCL22, and NF-κB activation associated with M2 differentiation (39, 42, 43). M2 GAMs have been shown to mediate immunosuppression within the TME and promote tumor progression (44, 45). Activation of MerTK, a receptor tyrosine kinase, polarizes GAMs to an immunosuppressive phenotype, but inhibition of MerTK from GAMs decreases immunosuppressive CD206^+^ GAM phenotype, leading to prolonged survival in GBM mouse models (**Figure 1B**) (46).

Several studies have advocated that pro-inflammatory M1 phenotype—associated with upregulation of CD115 and Siglec and consecutive production of IL-1β, IL-6, or IFN-γ—is critical for tumor eradication (39), especially since M1 phenotype has been reported to correlate with favorable survival outcomes in many human cancers (47). However, this trend appears inconsistent in glioma as inhibitory immune checkpoints such as PD-1, CTLA-4 and TIM-3 are consistently upregulated in M1/M2 GAMs which have been shown to significantly decrease patient prognosis (48, 49). Mice study has revealed that disruption of GBM-derived IL-6, known to induce myeloid PD-L1, reduces local and systemic myeloid-driven immunosuppression (50). In addition, GAMs in the TME are highly heterogeneous, with dynamic phenotypes and functions that are continuously shaped in response to the tumor. The binary M1/M2 classification appears too simplistic to explain the phenotype and functions of GAMs in tumors; dimensional data analysis from scRNA-seq reveals that GAMs possess multi-genomic phenotypes that encompass various M1 and M2 genes (41). Blood-derived macrophages, more so than resident microglia, have been reported to upregulate immunosuppressive cytokines and display an oxidative metabolism of M2 phenotype in the glioma microenvironment (41). A recent study further demonstrated regional differences of inflammatory responses in GAMs. GAMs in tumor core evolve toward pro-inflammation and are negatively correlated with PD-1 signaling, whereas GAMs in tumor periphery evolve toward anti-inflammation (37).

It has been previously recognized that GAMs undergo constant epigenetic and metabolic reprogramming regarding oxidative phosphorylation and anaerobic glycolysis, swinging between pro- and anti-inflammatory responses for growth-promoting or tumor-killing activity (51–54). Anti-inflammatory GAMs use the tricarboxylic acid (TCA) cycle in mitochondria to produce electrons that are essential for oxidative phosphorylation of glucose to generate high amounts of adenosine triphosphate (ATP) (55). This process fuels the mitochondrial electron transport chain and generates ROS, NADPH, and NO (56). Pro-inflammatory GAMs tend utilize anaerobic glycolysis, converting pyruvate into lactate (54, 57). Increased levels of lactate and TCA intermediates further upregulate histone hyperacetylation for IL-1β, TNF-α and IL-6 gene transcription (58, 59). In addition, gliomas are discovered to facilitate metabolic reprograming driven by mutations in the genes for the isocitrate dehydrogenase (IDH) and receptor tyrosine kinase (RTK) pathways (60). At present, even though signature mutations in known metabolic enzymes are recognized as being important, the metabolic landscape of gliomas is not incorporated with GAM pro- and anti-inflammatory environmental cues and patient prognosis.

## Glioma/Tumor-Infiltrating Neutrophils

Neutrophils with short life span are the most populous circulating leukocytes (61). In contrast to macrophages, neutrophils were traditionally considered bystanders in the TME. However, recent studies have uncovered distinct capabilities of neutrophils throughout each step of carcinogenesis from tumor initiation to primary tumor growth to metastasis. The degree of neutrophil infiltration in gliomas is significantly correlated with pathologic grade (62). Recently, new tools for genetic analysis further discovered the importance of tumor-associated neutrophils in the TME (63, 64). Thus, the efficacy of either traditional or novel strategies for treating cancers is likely determined by the phenotype of neutrophils in TME (61, 65).

In general, neutrophils play complex roles in tumor progression and metastases. Neutrophils are polarized to anti-tumor (N1) phenotype with IFNβ signaling or pro-tumor (N2) phenotype with TGFβ signaling in the TME (66, 67). This diversity of neutrophil behavior includes polar opposite functions in mediating tumor immunity (68). Additionally, TAMs and tumor-infiltrating lymphocytes (TIL), which are critical components in the TME, can be modulated by neutrophils to influence tumor development and T cell-dependent antitumor immunity. Neutrophils can be reprogrammed to express pro-tumor phenotype from intrinsic anti-tumor activity when recruited to the tumor (from N1 to N2) (66, 68). The N2 TANs can then facilitate tumor growth by suppressing T cell immunity and inducting genetic instability, tumor cell proliferation, angiogenesis, and metastasis (**Figure 1C**). Production of ROS and the release of microparticles (microRNAs miR-23A and miR-155) by neutrophils can downregulate molecules that maintain nuclear integrity and further lead to genetic instability (69–71). In addition, the epidermal growth factor (EGF), hepatocyte growth factor (HGF) and platelet- derived growth factor (PDGF) produced by neutrophils can facilitate tumor progression (72, 73). Neutrophil elastase (NE) and matrix metalloproteinase 9 (MMP9) (secreted by neutrophils) that cleaves laminin 111 (74, 75) lead to trigger cancer cell proliferation *via* activation of integrin signaling (74). In addition, pro-angiogenic factors including BV8, S100 proteins (S100A8, S100A9), and MMP9 released by neutrophils lead to activation of vascular endothelial growth factor A (VEGF A). Thus far, most studies demonstrated protumor roles of neutrophils (76, 77). Therefore, targeting TANs in immunotherapy for cancers, especially reprogramming of neutrophils from pro-tumor to anti-tumor phenotypes, holds promise to improve the efficacy of cancer treatments and possibly become the next-generation immunotherapy (78). Preclinical studies have shown positive results combining neutrophil depletion (anti-Ly6G antibody) and anti-PD-1 antibody treatment on glioma bearing mice (79). Additionally, there are currently several clinical trials targeting neutrophils. For example, clinical trials of Galunisertib (TGFβ receptor 1 kinase inhibitor) alone or combined with lomustine or temozolomide are ongoing in patients with recurrent glioblastoma (NCT01582269, NCT01682187, NCT01220271).

Neutrophil extracellular traps, NETs, are formed in response to extracellular pathogens and are specialized formation of fibrous decondensed chromatin with associated histones, MPO, and various cytoplasmic proteins, such as neutrophil elastase, cathepsin G, and lactoferrin. NETs have been shown to have diverse roles. Aggregated NETs can isolate immunostimulatory materials, which leads to limited immune activity and inflammation (80–82), but high density of NETs can cause organ or tissue damage (80, 83, 84). However, the role of NETs in the TME is an interesting unknown for cancer researchers. In theory, NETs can trap cancer cells and facilitate cytotoxic effects using ROS (84, 85). However, NETs can promote cancer metastasis by isolating circulating tumor cells (86). In glioma cells, NETs are thought to induce the IL-8 expression, which is correlated with tumor burden and prognosis through a HMGB1- and RAGE/ERK/NF-kB axis-dependent manner (**Figure 1C**) (87). Furthermore, glioma cells can induce NETs formation *via* IL-8 production by glioma (87). T-cell immunoglobulin and mucin domain-3 (TIM-3) interact with HMBG1 in TADCs and have an inhibitory, antitumor effect (88–90). A recent study has reported that TIM-3 can suppress the uptake of extracellular DNA in TADCs, which may influence the NETs production (91). Toll-like receptor 2, one of the HMGB receptors, is believed as correlation with NETs production, and HMGB1-mediated TLR2 signaling plays a critical role in eliciting glioblastoma regression, However, further studies are still needed to clarify the protumor and antitumor functions of NETs in glioma.

## Glioma/Tumor-Infiltrating Dendritic Cells

Dendritic cells (DCs) are professional antigen-presenting cells in the myeloid lineage that signal with and activate CD4^+^ and CD8^+^ T lymphocytes and natural killer (NK) cells to target the specific antigens presented (22, 92, 93). Since DCs usually confer protection against pathogens and disease, they are critical mediators of anti-tumor immunity and can be pulsed with peptide epitopes of tumor antigens to prime CD8+ T cells for an anti-tumor response (94). With induced activation and IL-12 signaling, mature GADs can then activate T lymphocytes against tumor antigens, even in the presence of the immunosuppressive TME induced by TGF-β2 signaling (95). However, DCs are manipulated by tumors to promote tumor growth and cancer disease progression.

There are many signaling pathways and secretory factors in the glioma TME that promote tumorigenesis (**Figure 1D**). STAT3 signaling in mouse glioma tumor-associated myeloid progenitor cells induces S100A8 and S100A9, which are inflammatory factors that arrest myeloid cell maturation, including DCs (19). This leads to a decrease in tumor-infiltrating and GADCs in the TME as well as the peripheral blood circulation, leading to a cyclic effect culminating in widespread immune suppression, a condition that favors tumor growth (19, 22, 96). CXC chemokines are also used by glioma cells to manipulate DC-mediated T cell immunity (96). CXCL1 and CXCL8 are enhanced in GBM patients, biomarkers for poorer prognosis, and positively correlated with DC and negatively correlated with CD8^+^ T cell infiltration in the TME (97). GADCs, along with FGL2 and CCL2 expressed by tumor cells and GAMs, also induce T_regs_ to suppress anti-tumor responses by inhibiting DC antigen presentation (22, 96). Furthermore, macrophage migration inhibitory factor (MIF) correlates with decreased GAD migration as well as decreased maturation, likely contributing to the tumor-tolerant immune state observed in GBM (98).

Vascular Endothelial Growth Factor (VEGF) is another immune-modulating factor expressed and secreted by tumor cells that acts as a double-edged sword. It promotes tumor growth *via* angiogenesis and inhibits GADC maturation by downregulating costimulatory factors CD80 and CD86, which are necessary to produce robust anti-tumor immune responses (**Figure 1D**) (5, 8). Glioma cells that express VEGF also have altered metabolomes (96). Upregulated hexokinase 2, phosphoglycerate dehydrogenase (PGHDH), 3-hydroxy-3-methylglutaryl-CoA reductase (HMGCR), cyclooxygenase 2 (COX-2), prostaglandin E synthase (PGES), and mutated IDH1 and IDH2 increase VEGF expression, which exacerbate suppressive effects on GADCs (96). Altered glycolysis and lactic acid homeostasis in glioma cells further contribute to tolerant GADC phenotypes (96, 99, 100). Upregulated glycolytic enzymes and GLUT1/3 transporters increase lactic acid uptake by GAMs and GADCs, which contributes to expression of inhibitory phenotypes (96). Additionally, expression of indoleamine 2,3-dioxygenase (IDO)-1/2 by glioma cells has been shown to be proportional to tumor grade; IDO is expressed by DCs within the TME and helps to recruit T_regs_, which further exacerbate and maintain the immunosuppressive state (96).

An additional subset of human DCs include plasmacytoid DCs (pDCs), which normally produce type 1 interferons in response to viral infections (101). However, in glioma patients, IFN-α and TLR7/9 signaling are downregulated, leading to a TME favoring tumorigenesis and a tolerogenic T cell response (101). pDCs have been previously shown to be the major subtype of DCs and antigen presenting cells at-large in glioma models and help recruit T_regs_ to the TME *via* TGF-β, secreted by glioma cells (101),. CXCL9, CXCL10, and CXCL12 signaling by glioma cells also help recruit pDCs to the TME (101). This flexibility in function—ranging from immunogenic to tolerogenic—makes this subset of DCs particularly exploitable by tumors for establishing an immunosuppressive TME, but it also makes them potential targets for effective anti-tumor therapies (101).

Because DCs can be manipulated to induce anti-tumor immunity, there have been many investigations and clinical trials for DC vaccine treatments. A meta-analysis of several phase II DC vaccine clinical trials revealed significant increases in overall and progression-free survival for GBM patients receiving DC vaccines in addition to standard of care (surgery, chemotherapy, and radiation therapy) (102). Research on the mechanisms of immunogenic cell death-based DC vaccines showed that their efficacy relied upon ROS and danger signals stimulated by the vaccine, as well as functioning DCs and CD8^+^ T cells (103). They also demonstrated that DC vaccines modified T cell homeostasis in the TME from the immune tolerant or suppressive T_regs_ to T_H_1, T_H_17, and cytotoxic CD8^+^ T cells that mediate anti-tumor immune responses, even overcoming immune disruptions caused by chemotherapy (103). There are also promising preliminary results from the first phase III trial of DC vaccines against GBM (104). Though there was crossover in the treatment design, so that about 90% of the intent-to-treat population eventually received DCVax-L, median overall survival (mOS) was 23.1 months after surgery—increased to 34.7 months for MGMT methylated patients—and there was also a group with extended survival (mOS of 40.5 months) (104). While the mOS for MGMT unmethylated patients was approximately 19 months, these mOS are improvements compared to the standard 15-16 months (105). These developments show exciting promise for immunotherapies for GBM patients that target and manipulate DC functions and interactions.

## Conclusions

In the past decades, emerging evidence showed the important role of myeloid cells in TME through great progress in fundamental and translational researches. Macrophages, neutrophils, and DCs have the dual functions of both pro-tumor and antitumor phenotypes within the TME, and this diverse function is probably a reflection of their plasticity in response to environmental cues. Therefore, based on understanding complex interaction between immune cells and glioma cells, various immunotherapeutic approaches, especially combination strategies, have been investigated and shown efficacious against glioma in some preclinical studies. However, conflicting research findings indicate the necessity of performing additional studies to assess efficacy in specific patient groups.

## Author Contributions

Y-JL, CW, and JW wrote the manuscript. Y-JL drew the figures. ML initiated the concept and supervised the writing. All authors contributed to the article and approved the submitted version.

## Conflict of Interest

ML has received research funding from Arbor, BMS, Accuray, Tocagen, Biohaven, Kyrin-Kyowa, Biohaven, Urogen. He also has been a consultant for Tocagen, VBI, InCephalo Therapeutics, Pyramid Bio, Merck, BMS, Insightec, Biohaven, Sanianoia, Hemispherian, Black Diamond Therapeutics, Novocure, Noxxon, and a shareholder of Egret Therapeutics.

The remaining authors declare that the research was conducted in the absence of any commercial or financial relationships that could be construed as a potential conflict of interest.

## Publisher’s Note

All claims expressed in this article are solely those of the authors and do not necessarily represent those of their affiliated organizations, or those of the publisher, the editors and the reviewers. Any product that may be evaluated in this article, or claim that may be made by its manufacturer, is not guaranteed or endorsed by the publisher.
